# Cardiac interoception in the museum: A novel measure of experience

**DOI:** 10.3389/fpsyg.2024.1385746

**Published:** 2024-06-19

**Authors:** Emma S. Stephenson, Kenneth Koltermann, Gang Zhou, Jennifer A. Stevens

**Affiliations:** ^1^Department of Psychological Sciences, College of William & Mary, Williamsburg, VA, United States; ^2^Department of Computer Science, College of William & Mary, Williamsburg, VA, United States

**Keywords:** interoceptive ability, interoceptive accuracy, cardiac interoception, interoceptive awareness, aesthetic appreciation

## Abstract

Interoception is the perception of the body’s internal signals in response to various external and internal stimuli. The present study uses a novel method adapted from the CARdiac Elevation Detection Task to examine cardiac interoception objectively and subjectively in a unique context—in the presence of art. Self-report questionnaires were used to measure subjective interoceptive awareness, subjective interoceptive accuracy, and aesthetic appreciation. For objective interoceptive accuracy and sensibility, a wearable device (Shimmer) measured heart rate (HR) and connected to a mobile application to prompt two questions: “Is your heart beating faster than usual?” and “How confident are you in your previous response?” Participants explored an art gallery for 40 minutes while the Shimmer measured their HR and randomly prompted them to answer the questions. Using a Generalized Estimating Equation model, interoceptive sensibility was not found to predict the odds of submitting a correct response. It was also found that art does not improve participants’ perceptions of their HR. Finally, there was no relation between aesthetic appreciation and subjective or objective cardiac interoception. Despite lack of statistical significance, the current study’s method presents an improved method by examining interoceptive accuracy in the moment under ecological conditions. To date, findings and methods used in interoception are inconsistent or flawed; the value in the current study lies in the development and demonstration of a method to examine how the environment influences the body and self-awareness across a wide variety of contexts, thereby offering a possible standardized measure of interoception for investigators to adopt.

## Cardiac interoception in the museum: a novel measure of experience

### What is interoception

Interoception, at the conscious level, is the perception of the body’s internal signals in response to various internal and external stimuli. Outside our consciousness, interoception describes the brain’s automatic formation of probabilities for sensory experiences within the body, given past experiences (Friston, 2010; Clark, 2013). As interoception research grows, new definitions arise to consider the multidimensionality of interoceptive abilities, from perceiving hunger and temperature to heart rate (HR) and metabolism (Khalsa and Lapidus, 2016). While Vaitl (1996) describes interoception as generally relating to proprioception and visceroception, Paulus (2013) defines interoception as the integration of information in the body with the central nervous system. There is no consensus on whether interoception should be measured in terms of attendance to internal signals (attention or awareness), perceived intensity of internal signals (magnitude), how well an individual interprets these signals (accuracy), believes they interpret sensations (sensibility), or views perceptions of the body as helpful or harmful (beliefs; Schandry et al., 1993; Simmons et al., 2012; Khalsa et al., 2015; Forkmann et al., 2016; MacCormack et al., 2022).

One approach utilized to explain interoception is allostasis, which describes the brain as a predictive organ that anticipates the body’s needs (Barrett and Simmons, 2015). Predictions based on probabilities from past experiences serve as hypotheses that the brain refines as sensory input is received. The brain does not merely react but anticipates information from the world and within the body, allowing for refined energy allocation. Prediction errors allow for the refinement of active inference, resulting in the production of more accurate visceromotor predictions. The body’s visceral experience is a combination of what is remembered of past and expected sensory experiences.

Changes in the body and brain influence emotion, yet we are not always perceptive of these physiological signals. Anger because of hunger, for example, is not always recognized by the “hangry” individual, and perception of emotion may not be correctly attributed to the body but other noise (MacCormack and Lindquist, 2016). One person may be sensitive to the somatic markers that assist emotion perception; however, another individual may experience elevated HR and sweaty palms yet not pay attention to these biomarkers (Bechara and Damasio, 2005). Somatic markers create unconscious changes in physiology that influence emotion and consequent decisions, reflective of interoceptive processes that are not perceived despite the body’s attempt to tell us (Bechara et al., 2005). Attention to these signals is associated with more intense emotional experiences that influence cognitive processes for better or worse, whether those be biases, judgments, or behaviors (Damasio et al., 1991; Garfinkel and Critchley, 2013).

Some individuals are not acute interoceptors and attribute decisions to “a gut feeling,” whereas others flag changes in physiology to explain attitudes. Being consciously interoceptive, termed *interoceptive ability*, allows one to interpret and regulate their body’s needs in response to hindrances to functioning. Possessing interoceptive ability has been examined in relation to various physiological and psychological experiences, such as those involving the gastrointestinal tract, hunger, eating disorders, stress, and cravings (Young et al., 2017; MacCormack and Lindquist, 2019; Khalsa et al., 2022). Increasing links to health illustrate the benefits of conscious interoceptive insight (Khalsa et al., 2018; Adams et al., 2022). Poor interoception, related to poor emotion regulation, contributes to the development of mental disorders, and individuals with depression or anxiety score lower on measures of *interoceptive accuracy* (IAcc) in comparison to non-clinical controls, such as the Heartbeat Counting Task (HCT) and Heartbeat Detection Task (HDT; Paulus and Stein, 2010; Herbert and Pollatos, 2014; Mata et al., 2015; Quadt et al., 2018; Martin et al., 2019).

### Measuring interoception

About a third of individuals are cardiac interoceptive, perpetuating the need for methods that accurately measure HR detection (Khalsa and Lapidus, 2016). Various methods attempt to measure *cardiac interoception*, such as the HCT, which requires participants to count their heartbeats in timed intervals, or the HDT, which asks participants to synchronize a tone to their HR. Self-reports are then compared to an objective measure of HR to determine accuracy (Leganes-Fonteneau et al., 2021; Desmedt et al., 2022; Legrand et al., 2022). However, these tasks are flawed and likely invalid (Zamariola et al., 2018). The HDT fails to discriminate between *interoceptive awareness* (IAw) and IAcc. Murphy et al. (2019) describe IAcc as the ability to interpret signals correctly yet IAw as a metacognitive capability determined by correspondence between IAcc and how well an individual believes they perceive their body’s signals (i.e., interoceptive insight). Second, these tasks are vulnerable to bias, such as prior knowledge of resting heart rate (RHR; Windmann et al., 1999; Brener and Ring, 2016). Further, these tasks measure cardiac interoception during rest. Understanding interoception in contexts of discomfort (e.g., anxiety, hunger, dietary fasting) is significant for understanding the relationship between interoception and health (Khalsa et al., 2018; Rominger et al., 2021). Another limitation is distinguishing between subjective and objective measurement. Murphy et al. (2019) present a 2 × 2 model of interoceptive abilities to clarify if a measure is objective or subjective, as well as if it measures accuracy or attention (awareness). While some subjective measures of interoception specify which facet of interoception they measure (e.g., the Body Perception Questionnaire measures awareness), many do not.

### Why examine art

Inspired by the CARdiac Elevation Detection (CARED) Task (Ponzo et al., 2021), the present study compares subjective ratings of IAcc to an objective measure of HR—though in the presence of art. Research examining the influence of art on cognition and perception (i.e., neuroaesthetics) has continued to grow since the 1960s when visual arts were the most common medium used to study aesthetics (Arnheim, 1966; Berlyne, 1971). Various factors contribute to aesthetic experiences, one being *aesthetic appreciation*. Curiosity for and enjoyment of music are related to individual differences in musical training, for example (Gerstgrasser et al., 2023). Although no research directly examines aesthetic appreciation and interoception, those trained in the arts are more interoceptive, suggesting a relationship between aesthetic interest and interoception (Christensen et al., 2017). Other research not specifically examining interoception finds that individuals considered experts in music experience more intense and diverse emotion when listening to music in comparison to non-experts (Gerstgrasser et al., 2023). Similarly, individuals with arts education experience heightened feelings of liveliness when viewing art (Miu et al., 2016). Given that emotion influences and is influenced by internal perceptions, aesthetic appreciation may moderate the relationship between emotion reactivity and interoceptive ability in the presence of art. Overall, research suggests that individuals with art training are more interoceptive and experience greater emotional responses during aesthetic experiences, which relate to greater physiological arousal. Further, greater physiological intensity is associated with heightened ability to notice changes in physiology (Ring et al., 2015; Körmendi et al., 2021), supporting our hypothesis that individuals reporting greater aesthetic appreciation are more interoceptive.

A contributor to increased interoceptive ability in those engaged in the arts may be reduction in the stress hormone cortisol. Previous research finds that gallery visits as short as 35 min significantly reduce cortisol and reports of stress, identifying cortisol as a primary contributor to dysregulated interoception (Clow and Fredhoi, 2006; Schulz and Vögele, 2015). Potentially, those with greater aesthetic appreciation are inclined to engage in aesthetic experiences and, therefore, experience reduced cortisol that improves bodily perceptions. Therefore, it may be that individuals that report greater aesthetic appreciation will perform better in a task that objectively measures their IAcc. Given art reduces cortisol, which inhibits interoception, we hypothesize that as time progresses in the presence of art, the odds of correctly perceiving one’s HR will improve.

When participants explore galleries unrestricted, self-reports of aesthetic-emotional experience significantly relate to changes in HR intensity, providing evidence of the power aesthetics have on the body and emotion (Tschacher et al., 2012). In-museum data collection is a new and promising method for capturing physiological change under ecological conditions where greater physiological arousal occurs in contrast to viewing artwork in the lab (Mastandrea et al., 2018; Krauss et al., 2021). Recent research examining aesthetic experience has measured physiological responses during live concerts, for example (Wald-Fuhrmann et al., 2021; Czepiel et al., 2023; Wald-Fuhrmann et al., 2023). Although experience sampling introduces a lack of controls, museums present a multi-sensory experience that extends the applicability of results to where they are most helpful (i.e., outside the lab).

### Purpose of the present study

In addition to examining the relationship between interoception and aesthetic appreciation, this study introduces a novel measure of interoceptive ability to further understanding of differences between subjective and objective measures of interoception, as well as between IAw and IAcc. The current study examines self-reports of physiological responses to an aesthetic experience in a naturalistic setting, a growing concept of data collection (Bannister and Eerola, 2023; Merrill et al., 2023).

Using the current study’s novel method, we are first interested in whether results obtained from the CARED Task are replicable. The CARED Task is a recent measure of interoceptive ability by asking participants to report their HR in the moment and outside the lab. We expect to replicate results from Ponzo et al. (2021) and find no significant relationship between objective IAcc (measured by the number of correct responses submitted by the participants during the task) and subjective IAcc [measured with the Interoceptive Accuracy Scale (IAS)]. If the current study obtains this result, there is further evidence that subjective measures of interoception are not measuring objective interoceptive ability. This result, as found by Ponzo et al. (2021), contrasts with Garfinkel et al. (2015) and Murphy et al. (2019), who obtained significant positive correlations between the IAS and objective IAcc with the HCT.

Similarly, we expect no relationship between objective IAcc and subjective IAw, measured by the Body Awareness (BA) subscale of the Body Perception Questionnaire Short Form (BPQ-SF). Our hypothesis is supported by current literature, and we expect our novel method to obtain a similar result, given argued reliability of past measures (Critchley et al., 2004; Garfinkel et al., 2015; Ferentzi et al., 2018). If the current study’s method replicates these results, there will be further evidence of significant differences in subjective and objective measures yet also awareness and accuracy. Regarding the BPQ and IAS, previous literature finds no relationship between these subjective measures of IAw and IAcc, and therefore we expect the same result (Murphy et al., 2020; Ponzo et al., 2021; Gabriele et al., 2022). However, we will utilize the BA subscale of the BPQ-SF to measure attention to physiological signals. The BPQ is a general measure of subjective IAw, yet there is internal reliability between its subscales (Cabrera et al., 2017).

Previous literature argues that participants’ confidence, or interoceptive insight, predicts objective IAcc (Khalsa et al., 2008). This is seen with the CARED Task and HCT (Murphy et al., 2020). We hypothesize a similar result where confidence will significantly predict the odds of submitting a correct response and positively relate to the number of correct responses. Given that the presence of art reduces cortisol, therefore improving interoception, we expect confidence to increase.

## Method

### Participants

Participants were recruited from the university’s research participation system and were at least 18 years old. The present study recruited 65 participants, and this sample size is based on previous literature (Ponzo et al., 2021; Schuette et al., 2021; Legrand et al., 2022). Of the 65 participants, four were excluded from analyses due to excessive noise or loss of data, resulting in a final sample of 61 (34 female, age range: 18–22, *M =* 18.98, *SD = 0*.892).

### Measures

#### Subjective interoceptive awareness

Subjective IAw is assessed using the BA subscale of the BPQ-SF (Porges, 1993). Participants reported their attention to internal body signals. The BA subscale comprises 26 items rated from Always (5) to Never (1), with scores ranging 26–130. Higher scores indicate greater subjective IAw.

#### Subjective interoceptive accuracy

Subjective IAcc is assessed using the IAS (Murphy et al., 2019). Participants reported how well they believe they perceive bodily sensations. The IAS comprises 21 items rated from Strongly Agree (5) to Strongly Disagree (1), with scores ranging 21–105. Higher scores indicate greater subjective IAcc.

#### Objective interoceptive accuracy and sensibility

A wearable device (Shimmer) recorded participants’ HR every 10 milliseconds during the gallery exploration. The Shimmer was selected due to its non-restrictive design (weighing 1.3 lbs) and validation of wearable sensors in biomedical research (Keogh et al., 2021; Singstad et al., 2021; Vijayan et al., 2021; Watson et al., 2021). The device was attached to an elastic strap that adjusted to the wrist, and an optical pulse sensing probe was worn on the index finger using Velcro, similar to a ring. A smartphone application was developed for Bluetooth communication with the Shimmer. The screen prompted participants to answer two questions via vibration at random times within five 8-min intervals: “Is your heart beating faster than usual?” (answered Yes/No) and “How confident are you in your previous response?” (answered 0–100). Participants were prompted randomly to prevent increased HR from anticipation or improvement due to routine practice.

When participants respond to the first question, subjective IAcc is initially captured; however, responses become a measurement of objective IAcc once compared to RHR to confirm if their perception is correct. Out of five, a final sum of correct responses constitutes a participant’s objective IAcc. The second question measures interoceptive sensibility.

#### Aesthetic appreciation

**Figure 1:**
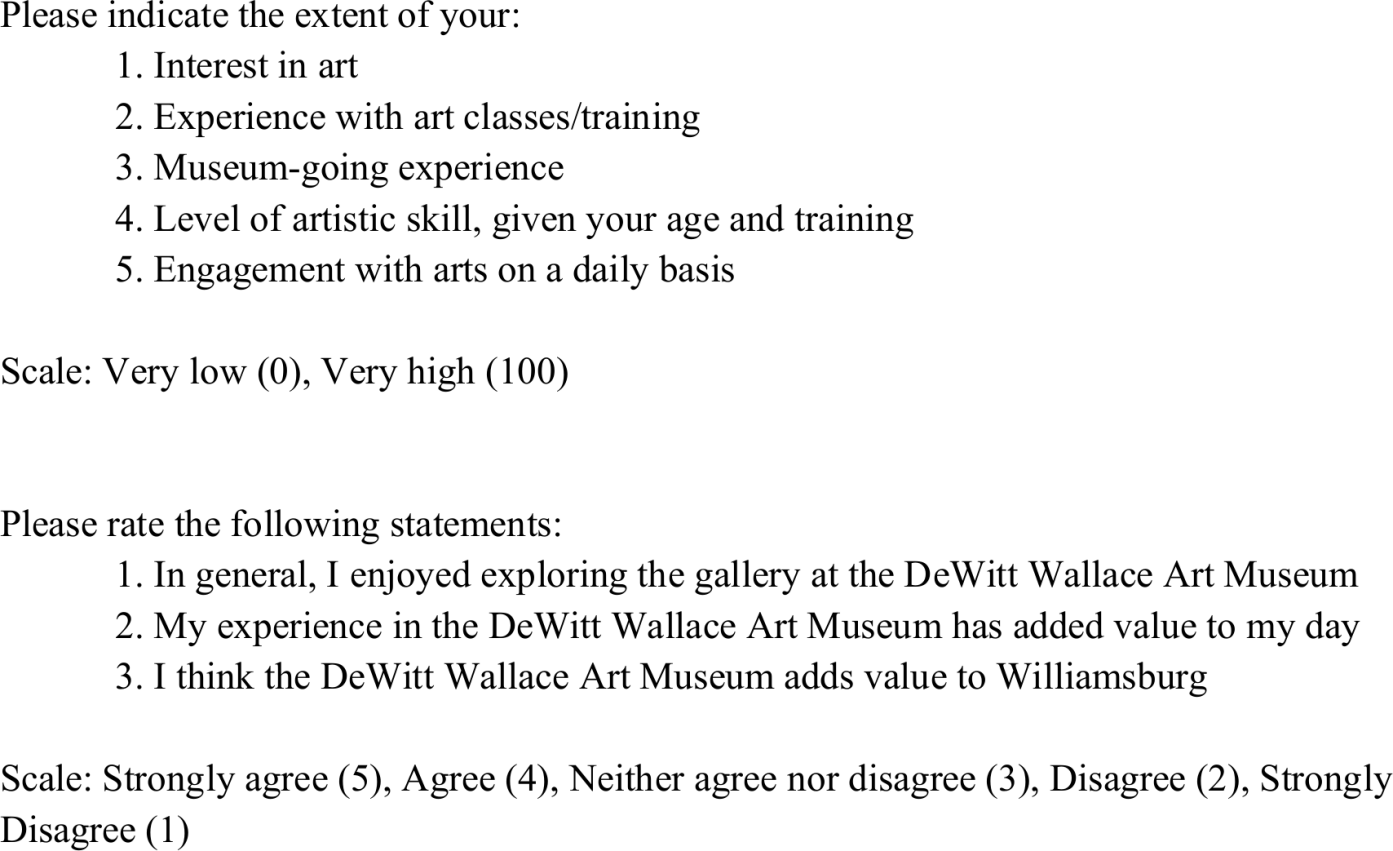
Aesthetic appreciation scale (AAS).

Aesthetic appreciation is assessed with a novel 8-item scale (*α* = 0.71) titled the Aesthetic Appreciation Scale (AAS). Participants report their experiences and interests in art (Figure 1). The AAS is composed of 5 items rated from Very High (100) to Very Low (0), with scores ranging 0–500. In addition to the original five items, we included three items (*α* = 0.83) that specifically examine museum experiences. Participants rated each of the three items from Strongly Agree (5) to Strongly Disagree (1), with scores ranging 3–15.

### Design and procedure

The study occurred in the DeWitt Wallace Arts Museum. Once the Shimmer was attached, the researcher instructed participants to freely explore the gallery for 40 min. The participants held a smartphone with the application that randomly prompted the two questions. The Shimmer measured participants’ HR during the gallery exploration. At the end of the exploration, the application paused data collection and notified participants to return to the researcher; participants then completed the questionnaires on a tablet. While participants were at rest, the Shimmer resumed data collection for 15 min to determine RHR. If the questionnaires did not fill the entire 15-min period, participants read a copy of the university’s magazine.

### Data analysis

To clean and analyze data, researchers used IBM SPSS Statistics for Windows 29.0.0.0. HR data was processed by band-passing with a Butterworth filter between 40 and 150 beats per minute (BPM) to remove low/high-frequency noise. To convert photoplethysmography (PPG) data to BPM, researchers used the window approach in the HeartPy framework, which ran peak detection over slices of time 1 min in length (van Gent et al., 2019a,b). Participants’ RHR was then calculated by averaging the final 15 min of HR data. To determine participants’ responses to the first question as correct, the times at which participants submitted responses were matched to the times that the Shimmer recorded their HR; responses were recorded as 0 for incorrect (false alarm/miss) or 1 for correct (correct rejection/hit). A participant’s HR was identified as higher than usual if their HR exceeded their RHR by 5 BPM. We chose a 5 BPM cutoff since this exceeds the average inter-individual variability in RHR for adults and is described as non-normal (Quer et al., 2020).

#### Analyzing objective IAcc with a single score

The researchers summarized objective IAcc into a single score for each participant for Pearson correlations, which ranged from 0–5 to represent the number of correct responses.

#### Analyzing objective IAcc with a generalized estimating equation model

We created a Generalized Estimating Equation (GEE) model, which is the most appropriate statistical procedure given our hypotheses and non-normal data (Lipsitz et al., 1994; Landerman et al., 2011). Liang and Zeger (1986) and Zeger and Liang (1986) developed GEEs to analyze the influence of variables on a binary dependent variable (DV) across time and within subjects. To determine if the presence of art improves objective IAcc, our GEE considers change over time while including confidence as a covariate since it is reported with each response. Additionally, we hypothesize that as time with art increases, confidence will increase; therefore, we consider an interaction between confidence and time. A GEE is able to test our main and interaction effects with both categorical and continuous variables.

The GEE model predicts the odds of submitting a correct response by including confidence in each response, sequential time intervals, and an interaction between confidence and which interval the response was made. As instructed by Stokes (1999) and Ballinger (2004), the researchers specified the link function, shape of the DV’s distribution, and correlation structure (McCullagh and Nelder, 1989; Pindyck and Rubinfeld, 1990; Fitzmaurice et al., 1993; Fitzmaurice, 1995; Pan, 2001; Agresti, 2002; Harrison, 2002; Liu and Colditz, 2016).

## Results

Correlations and descriptive statistics for variables are shown in Table 1. Three participants did not complete the AAS, resulting in different degrees of freedom. There was no significant, positive relationship between the number of correct responses and IAS [*r*(59) = −0.07, *p* = 0.58], aesthetic appreciation [*r*(56) = 0.02, *p* = 0.12], or confidence [*r*(59) = 0.03, *p* = 0.79]. Further, we did not observe a significant, positive relationship between the IAS and confidence [*r*(59) = −0.10, *p* = 0.40], and aesthetic appreciation did not correlate positively to confidence, the IAS, BPQ-SF, nor BA subscale. As expected, there was no significant relationship between the BPQ-SF and the number of correct responses [*r*(59) = 0.15, *p* = 0.22] nor the IAS [*r*(59) = −0.18, *p* = 0.16]. However, when utilizing the BA subscale as a measure of subjective IAw, there was an unanticipated significant, negative relationship to the IAS [*r*(59) = −0.26, *p =* 0.03].

**Table 1:** Pearson correlations and descriptive statistics of all study variables.

Variable	1	2	3	4	5	6	7	*n*	*M*	*SD*
1. Number of correct responses ^a^	—							61	2.31	1.39
2. Average confidence ^b^	0.03	—						61	67.92	20.77
3. BPQ-BA	0.16	0.20	—					61	53.66	19.99
4. BPQ	0.15	0.16	0.95**	—				61	82.65	24.57
5. IAS	−0.07	−0.10	−0.26*	−0.18	—			61	46.36	8.45
6. Aesthetic appreciation	0.20	−0.10	0.11	0.12	−0.01	—		58	253.10	91.04
7. RHR	0.27*	−0.12	0.00	0.03	0.08	0.23	—	61	71.25	10.22

*p < 0.05. **p < 0.01. ^a^For the purpose of running Pearson correlations, the number of correct answers submitted in response to the first question (i.e., “Is your heart beating faster than usual?”) serves as a score for objective interoceptive accuracy. ^b^All five confidence ratings in response to the second question (i.e., “How confident are you in your previous answer?”) are averaged for each participant.

**Figure 2:**
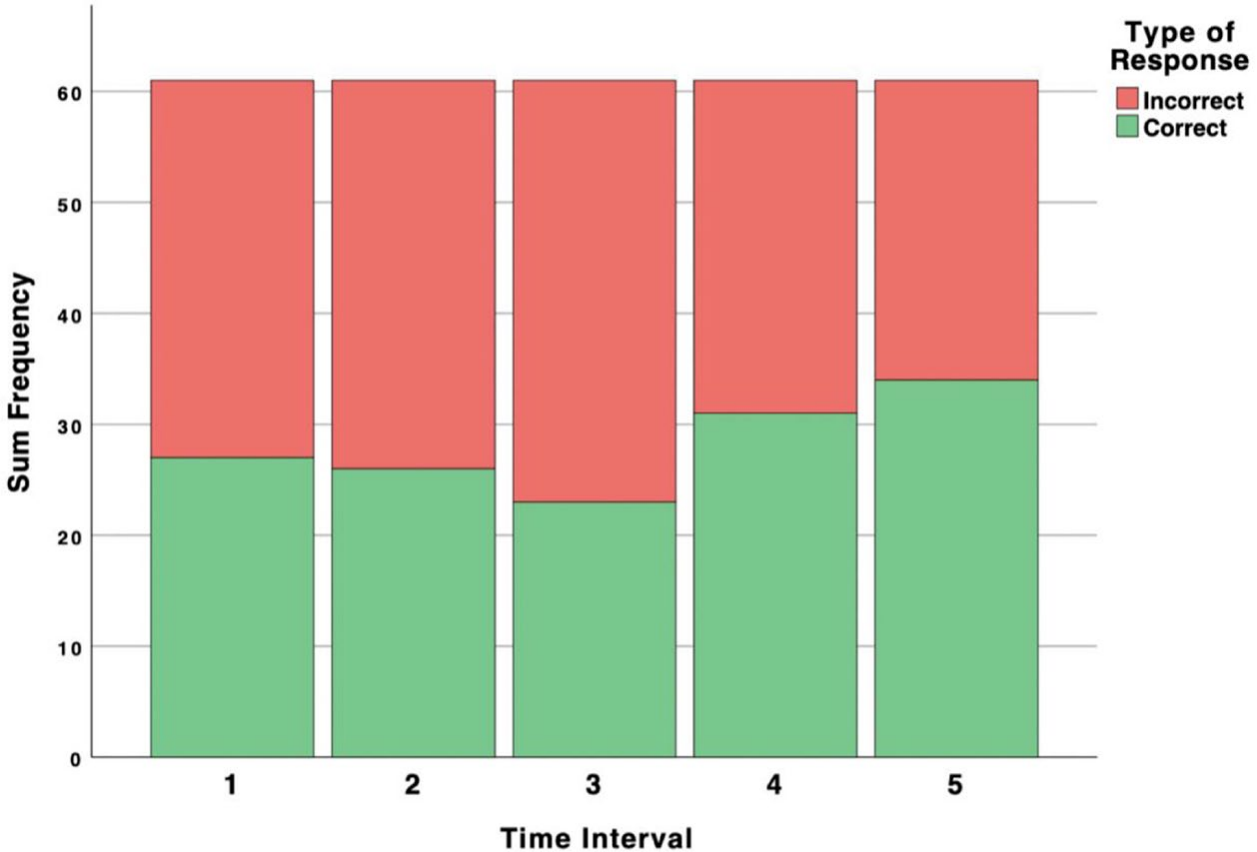
Ratio of correct and incorrect responses over time. The number of correct responses provided by each participant out of five is used as a measure of objective interoceptive accuracy, for the purpose of running Pearson correlations.

The ratios of correct–incorrect responses for intervals were fairly consistent. Following a decrease in the third interval, correct responses increase in the last two intervals (see Figure 2). The distribution for the number of participants submitting 0–5 correct responses is visually approximately normal, with most participants submitting 2 correct responses (4 participants submitting 0 correct responses, 15 submitting 1, 18 submitting 2, 13 submitting 3, 4 submitting 4, and 7 submitting 5). Averages for confidence in each interval are consistent over time, and we ran a repeated-measures ANOVA to examine confidence across the intervals. Mauchly’s test indicated that the assumption of sphericity had been violated, *X*^2^(9) = 22.13, *p* = 0.00, and degrees of freedom were corrected using Huynh-Feldt estimates of sphericity (∈ = 0.915). Confidence did not significantly increase as time in the museum progressed [*F*(3.66, 240) = 0.34, *p* = 0.83].

To determine the odds of submitting a correct response, the GEE model considered trial number, confidence, and an interaction between trial number and confidence. No significant model effects were found. Results of the GEE regression are presented in Table 2. The ratios of correct–incorrect responses reveal a pattern consistent with our hypothesis that objective IAcc will improve as time in the museum progresses (see Figure 2). In the first interval, participants respond below chance (34 incorrect–27 correct), yet this ratio gradually shifts toward improvement, as seen in the fourth interval (30 incorrect–31 correct), with participants scoring highest in the fifth interval (27 incorrect–34 correct), suggesting that performance may be expected to improve over time.

**Table 2:** GEE regression results with an autoregressive variance–covariance matrix.

Parameter	*β*	*SE*	95% Wald CI		Wald *X*^2^	Odds ratio	*p*
			*LL*	*UL*			
Intercept	0.15	0.64	−1.11	1.41	0.05	1.16	0.81
Trial 1	0.19	1.04	−1.84	2.24	0.03	1.20	0.85
Trial 2	−0.18	1.00	−2.16	1.79	0.03	0.83	0.85
Trial 3	0.49	0.96	−1.38	2.38	0.27	1.63	0.60
Trial 4	0.33	0.79	−1.21	1.89	0.18	1.39	0.66
Trial 5	0 ^a^	–		–	–	–	–
Confidence	−0.00	0.00	−0.02	0.01	0.41	1	0.52
Trial 1 × Confidence	0.00	0.01	−0.02	0.03	0.09	1	0.76
Trial 2 × Confidence	0.01	0.01	−0.01	0.03	0.66	1.01	0.41
Trial 3 × Confidence	0.00	0.01	−0.02	0.02	0.07	1	0.78
Trial 4 × Confidence	−0.00	0.01	−0.02	0.01	0.03	1	0.85
Trial 5 × Confidence	0 ^a^	–	–	–	–	–	–

CI, confidence interval; SE, standard error. ^a^Set to zero because this parameter is redundant.

## Discussion

The current study presents novel conclusions about the relationship between interoception and aesthetic appreciation and an improved measure for interoceptive ability. Our results reveal drastic distinctions between subjective and objective measures of interoception, suggesting that past studies utilizing subjective measures to draw conclusions about objective ability are potentially invalid. An individual may report themselves as interoceptive, or high confidence, yet perform poorly. Our results support criticisms that there is a significant distinction between IAw and IAcc, and that past studies examining interoception in terms of one but not the other fail to recognize interoception’s multi-dimensionality.

Pearson correlations confirm and contradict previous literature. Murphy et al. (2019) suggest the IAS may predict objective IAcc; however, we did not discover a significant, positive relationship between the IAS and the number of correct responses, which contradicts previous literature utilizing the HCT (Garfinkel et al., 2015; Murphy et al., 2020). Our result, however, is reflected in Ponzo et al. (2021), suggestive of a significant difference between objective and subjective IAcc, meaning we cannot rely on subjective measures to infer objective ability. We additionally did not find a relationship between subjective IAw, as measured by the BPQ-SF, and objective IAcc, which agrees with previous findings (Critchley et al., 2004; Garfinkel et al., 2015; Ferentzi et al., 2018; Ponzo et al., 2021). As expected and seen in previous literature, subjective IAw, measured by the BPQ-SF, did not significantly relate to subjective IAcc, measured by the IAS; however, IAS significantly related negatively to the BA subscale of the BPQ-SF, which was unexpected and requires investigation.

Unlike Ponzo et al. (2021), confidence did not predict the odds of submitting a correct response when made a main effect in the GEE model. When averaged per participant, confidence still did not correlate to the number of correct responses. Similar to Murphy et al. (2019), we did not find a significant relationship between the IAS and confidence. However, Murphy et al. (2019) found a significant, positive relationship between objective IAcc and confidence with the HCT, which we did not. These results are surprising given that previous literature argues that confidence reflects objective IAcc (Khalsa et al., 2008; Garfinkel et al., 2015).

We did not observe a significant relationship between aesthetic appreciation and the BPQ-SF, IAS, nor the number of correct responses, in contrast to previous literature. It may be that specific types of art and aesthetic experiences are responsible for the results we found. Some aesthetic experiences, such as concerts, create more intense physiological responses, and we know that as our HR increases, so does sensitivity to perceiving our HR correctly. A visual arts museum, however, causes a *decrease* in HR, making our task difficult (Merrill et al., 2023).

## Limitations and moving forward

The generality of subjective measures hinders the possibility of capturing a relationship between interoception and aesthetic appreciation. The diverse processes questioned in the BPQ-SF and IAS, from perceiving temperature of the ears to indigestion, sexual arousal to salivation, cloud detection of small effects. Researchers should examine specific types of interoception in relation to specific types of aesthetic appreciation. For example, an individual may possess an appreciation for concerts which may relate to cardiac interoception—or, an individual may be fond of ceramics, which may relate to skin-mediated interoception (Crucianelli et al., 2022; Crucianelli and Ehrsson, 2022). As we discovered, different results erupt when examining different subsets of interoception measures (i.e., the BA subscale versus the BPQ-SF; Vig et al., 2022).

Future studies should consider emotion at the time of HR responses. Emotion influences interoceptive ability and vice versa; consequently, we should consider how participants feel during an aesthetic experience. Previous research examining physiology during live concerts finds that emotions, and their intensities, provoked by art relate to enjoyment of experience (Svanås-Hoh et al., 2023; Tschacher et al., 2023). Possibly, the museum did not elicit intense emotional, and similarly physiological, responses, resulting in partially insignificant results.

## Conclusion

Interoceptive ability is flexible, capable of improving, which is beneficial in the context of art (Sugawara et al., 2020; Quadt et al., 2021). Art relaxes the body, and given interoceptive ability can be improved to perceive these physiological changes, it can be a resource for improving health. Art is a universal expression of the human experience made accessible to most populations and can function as a low-cost therapy. However, ability to improve health through perceptions of the body is limited by our assessments of interoception. While new measures, such as the CARED Task and the current study’s method, replicate some findings derived from previous measures, not all results are reproduced. There is a need to create, test, and re-test measures under ecological conditions (Open Science Collaboration, 2015; Garfinkel et al., 2022). Using smartphone applications to measure interoception, especially in environments where the body changes, is growing and encouraged (Plans et al., 2021). Interoception is a process that occurs at all times, and its measurement needs to extend to natural conditions to capture everyday perceptions. The current study moves us one step further by introducing a valuable interoception measure for researchers to adopt as research in this area continues to develop.

## Data availability statement

The datasets presented in this article are not publicly accessible. Requests to access the datasets should be directed to ES, estephensonwm.edu.

## Ethics statement

The studies involving humans were approved by The College of William & Mary Institutional Review Board. The studies were conducted in accordance with the local legislation and institutional requirements. The participants provided their written informed consent to participate in this study.

## Author contributions

ES: Conceptualization, Data curation, Formal analysis, Funding acquisition, Investigation, Methodology, Project administration, Resources, Software, Supervision, Validation, Visualization, Writing – original draft, Writing – review & editing. KK: Conceptualization, Data curation, Investigation, Methodology, Project administration, Software, Validation, Visualization, Writing – original draft, Writing – review & editing, Resources, Supervision. GZ: Data curation, Methodology, Resources, Software, Supervision, Writing – original draft, Writing – review & editing. JS: Conceptualization, Data curation, Formal analysis, Funding acquisition, Investigation, Methodology, Project administration, Resources, Software, Supervision, Validation, Visualization, Writing – original draft, Writing – review & editing.
